# Supplementary material for: Geospatial analysis of the associations between environmental contamination with livestock feces and children with chronic fascioliasis in the Anta province of Cusco, Peru
Source: PLoS Negl Trop Dis. 2022 Jun 16;16(6):e0010499. doi: 10.1371/journal.pntd.0010499 (PMC9242436; doi:10.1371/journal.pntd.0010499)
Supplement: S1 Table — (DOCX) [file pntd.0010499.s001.docx]

**SUPPLEMENTAL MATERIAL**

**Table S1:** “Number of livestock feces inside the 50 m buffer as per household status”

|  | Household Negative status | Household Positive status |
| --- | --- | --- |
| All negative feces (n) | 3428 | 292 |
| All positive feces (n) | 1590 | 146 |
| Negative cattle feces (n) | 1300 | 112 |
| Positive cattle feces (n) | 668 | 71 |
| Negative swine feces (n) | 1347 | 106 |
| Positive swine feces (n) | 336 | 35 |
| Negative sheep feces (n) | 781 | 74 |
| Positive sheep feces (n) | 586 | 40 |

Multivariate logistic regression at 50 meters: Variables were removed in the following order: sheep negative feces (p=0.9396), swine negative feces (p=0.7711), swine positive feces (p= 0.7968) to a p value < 0.05.
