# Supplementary material for: Geospatial analysis of the associations between environmental contamination with livestock feces and children with chronic fascioliasis in the Anta province of Cusco, Peru
Source: PLoS Negl Trop Dis. 2022 Jun 16;16(6):e0010499. doi: 10.1371/journal.pntd.0010499 (PMC9242436; doi:10.1371/journal.pntd.0010499)
Supplement: S2 Table — (DOCX) [file pntd.0010499.s002.docx]

**SUPPLEMENTAL MATERIAL**

**Table S2:** “Number of livestock feces inside the 100 m buffer as per household status”

|  | Household Negative status | Household Positive status |
| --- | --- | --- |
| All negative feces (n) | 7442 | 581 |
| All positive feces (n) | 3466 | 303 |
| Negative cattle feces (n) | 2820 | 226 |
| Positive cattle feces (n) | 1491 | 142 |
| Negative swine feces (n) | 3057 | 232 |
| Positive swine feces (n) | 690 | 70 |
| Negative sheep feces (n) | 1565 | 123 |
| Positive sheep feces (n) | 1285 | 91 |

Multivariate logistic regression at 100 meters: Variables were removed in the following order: sheep negative (p=0.9718), swine positive (p=0.8821), swine negative (p=0.3108), and cattle negative (p=0.0714) to a p value of < 0.05.
