# Supplementary material for: Geospatial analysis of the associations between environmental contamination with livestock feces and children with chronic fascioliasis in the Anta province of Cusco, Peru
Source: PLoS Negl Trop Dis. 2022 Jun 16;16(6):e0010499. doi: 10.1371/journal.pntd.0010499 (PMC9242436; doi:10.1371/journal.pntd.0010499)
Supplement: S3 Table — (DOCX) [file pntd.0010499.s003.docx]

**Table S3:** “Number of livestock feces inside the 200 m buffer as per household status”

|  | Household Negative status | Household Positive status |
| --- | --- | --- |
| All negative feces (n) | 17684 | 1372 |
| All positive feces (n) | 8720 | 752 |
| Negative cattle feces (n) | 6636 | 536 |
| Positive cattle feces (n) | 3725 | 340 |
| Negative swine feces (n) | 7350 | 562 |
| Positive swine feces (n) | 1845 | 166 |
| Negative sheep feces (n) | 3698 | 274 |
| Positive sheep feces (n) | 3150 | 246 |

Multivariate logistic regression at 200 meters: Variables were removed in the following order: sheep positive feces (p=0.9803), swine positive feces (p=0.8213), cattle positive feces (p= 0.7185), sheep negative feces (p=0.2158), and swine negative feces (p=0.0684) to a p value < 0.05.
