# Supplementary material for: Geospatial analysis of the associations between environmental contamination with livestock feces and children with chronic fascioliasis in the Anta province of Cusco, Peru
Source: PLoS Negl Trop Dis. 2022 Jun 16;16(6):e0010499. doi: 10.1371/journal.pntd.0010499 (PMC9242436; doi:10.1371/journal.pntd.0010499)
Supplement: S4 Table — (DOCX) [file pntd.0010499.s004.docx]

**SUPPLEMENTAL MATERIAL**

**Table S4:** “Adjusted univariate regression model for sheep positivity feces on household positivity status stratified by altitude of the household”.*

| Sheep positive | OR (95% CI) | P-value |
| --- | --- | --- |
| 50-m buffer lower altitudes | 0.19 (0.03-1.41) | 0.1052 |
| 50-m buffer higher altitudes | 0.90 (0.58-1.40) | 0.6443 |
| 100-m buffer lower altitudes | 0.90 (0.49-1.64) | 0.7255 |
| 100-m buffer higher altitudes | 0.98 (0.78-1.22) | 0.8548 |
| 200-m buffer lower altitudes | 0.90 (0.49-1.64) | 0.7255 |
| 200-m buffer higher altitudes | 0.98 (0.78-1.22) | 0.8548 |

- Model adjusted for: presence of poor sanitation, unsafe water intake, multiple household Fasciola infections.
