# Supplementary material for: Geospatial analysis of the associations between environmental contamination with livestock feces and children with chronic fascioliasis in the Anta province of Cusco, Peru
Source: PLoS Negl Trop Dis. 2022 Jun 16;16(6):e0010499. doi: 10.1371/journal.pntd.0010499 (PMC9242436; doi:10.1371/journal.pntd.0010499)
Supplement: S5 Table — (DOCX) [file pntd.0010499.s005.docx]

**SUPPLEMENTAL MATERIAL**

**Table S5:** “Adjusted Multivariate Logistic regression analysis stratified by higher or lower altitudes above the sea level associated with amount of livestock feces around different radiuses around the household”

| Buffer distance* | OR ( 95% CI) | P-value |
| --- | --- | --- |
| 50M less | None significant | n/a |
| 50M more   - Cow positive - Cow negative - Sheep positive | 2.22 (1.28-3.84)  1.68(1.16-2.43)  0.59 (0.35-0.97) | 0.0046  0.0058  0.0389 |
| 100 M less | None significant | n/a |
| 100 M more:   - Cow positive - Cow negative - Sheep positive | 1.51 (1.02-2.24)  1.35 (1.07-1.70)  0.71 (0.52-0.99) | 0.0370  0.0100  0.0446 |
| 200 M less | None significant | n/a |
| 200 M more:   - Cow positive - Cow negative - Sheep positive | 1.51 (1.02-2.24)  1.35 (1.07-1.70)  0.71 (0.52-0.99) | 0.0370  0.0100  0.0446 |

- Model included: cow positive/negative, sheep positive/negative, swine positive/negative, only significant values are shown to p < 0.05
- All models adjusted for poor sanitation, unsafe water use, multiple household Fasciola infections.
- Variables were removed in the following order:
  - 50M more: sheep negative (p=0.7546), swine negative (p=0.5893), swine positive (p=0.4073)
  - 100M more: swine positive (p=0.9283), sheep negative (p=0.8254), swine negative (p=0.4176).
  - 200M more: swine positive (p=0.9283), sheep negative (p=0.8254), swine negative (p=0.4176).
