# Supplementary material for: Geospatial analysis of the associations between environmental contamination with livestock feces and children with chronic fascioliasis in the Anta province of Cusco, Peru
Source: PLoS Negl Trop Dis. 2022 Jun 16;16(6):e0010499. doi: 10.1371/journal.pntd.0010499 (PMC9242436; doi:10.1371/journal.pntd.0010499)
Supplement: Attachment — Submitted filename: REVIEW RESPONSES PLOS NTDS 2022.docx [file pntd.0010499.s006.docx]

April 20, 2022

RE: Authors Response to Decision Letter for PNTD-D-21-01626.R1

Dear Editors,

We are submitting the revision made to the manuscript titled “Geospatial analysis of the associations between environmental contamination with Fasciola infected livestock feces and children in the Anta province of Cusco, Peru ” in response to the reviewers comments and suggestions. This manuscript was being considered for publication as original research in PLOS Neglected Tropical Diseases.

We would like to thank you and the reviewers for the comments and suggestions to improve the quality of our manuscript. We have diligently reviewed each comment and have addressed the concerns and suggestions made. This revised version of the manuscript has incorporated most of the suggestions made by the reviewers. Below you will find detailed responses to each reviewer's comments.

The manuscript has not and will not be submitted for publication elsewhere, so long as it is under consideration by PLOS Neglected Tropical Diseases.

Best Regards,

Miguel M. Cabada, MD MSc

Associate Professor

Division of Infectious Diseases

University of Texas Medical Branch

Director

Cusco Branch – Tropical Medicine Institute

Alexander von Humboldt

Universidad Peruana Cayetano Heredia

301 University Boulevard rt 0435

Galveston, TX, 77555

Telephone: +1 409 747 0223
email: [micabada@utmb.edu](mailto:micabada@utmb.edu)

**RESPONSES TO THE REVIEWERS COMMENTS:**

**METHODS:**

**REVIEWER 1**

- Feces and stool are both used in this paper. Pick one and be consistent in use.
  - Authors: The change was made as suggested. “Feces” is now used consistently throughout the manuscript.
- Lines 95-96. More detail is needed on how the three fecal samples were collected, especially from older children, and when this occurred in the context of a cross-sectional household visit. Were there repeat visits?
  - Authors: A description of the enrollment process has been included. Containers and instructions to collect stool samples were provided to each child at pre-school and schools. Freshly produced stool samples were collected the next morning in consecutive days. Children that failed to produce a stool sample were given instructions again to collect a sample the next day. Each child’s household was visited within 2 weeks of stool testing. The houses were visited once, and all animal samples were collected during the visit.
- Line 98. My understanding is that children were enrolled, then had 3 stools collected and tested. However, line 98 suggests that this study was instead a two-staged process with pre-testing of lots of kids to identify “households with multiple children infected with Fasciola”, followed by some sort of secondary enrollment of “only one infected child selected to participate while other children in the household with or without the infection were excluded.” By default if you are testing child stool for Fasciola, then they are participating. Please revise to improve clarity.
  - Authors: This cross-sectional study enrolled all children in pre-schools and schools in the targeted communities. Regardless of infections status, all children’s households were visited and when livestock were own at the household, manure was collected for testing. Multiple children from a household were eligible to participate in the study. All data was collected in a single stage as described in the answer to the previous comment by this reviewer. Children infection status was used to define the infection status of the household. The analysis unit for this study was the household not the children. We have clarify the definitions and the analysis to avoid confusion. We have also remove some of the information about individual children (e.g. age) and left the information pertaining to the households (e.g. socioeconomic score).
- Lines 100-102. Are these animals of the participating household or potentially those of a neighbor? Also, were any criteria used to eliminate animal stool that seemed old or desiccated?
  - Author: Only fresh animal stools around each household were collected. Stool had to be moist and “soft” (depending on the species) for collection. The animal stool specimens closest to the household were collected. It is not possible to ascertain that all the stool collected belonged only to animals owned in that household. However, it would be highly unlikely that the neighbor’s livestock would be that close to the living space. We have included a bird’s view of the location to increase comprehension.
- Line 108. “Children’s” or “Child”
  - Authors: The change was made as suggested.
- Lines 113-114. Related to my comment about lack of clarity on study enrollment, the difference between “Chronic fascioliasis” and “Positive Fasciola household” outcomes is not clear from the study design, and the individual-level fascioliasis outcome is not reported or analyzed as an outcome anywhere in the analysis, although it should be.
  - Authors: Please see above for an explanation of the definition and classification of households. Children with chronic fascioliasis was defined as those having eggs in stool in at least one stool microscopy test. A positive Fasciola household was considered the analysis unit and defined as a home with at least one child with chronic Fasciola infection. The individual child’s data has not been reported or removed to avoid confusion.
- Lines 124-131. Why are you using backward regression modeling with only four (three in practice) variables included in the model? If this was truly the process, then you need to provide the full stepwise rules you used for evaluating model fit at each stage of model simplification. Related to this and my comment above, why has the model not also been adjusted for multi-infections among household members and proximity of water bodies with snails? Children could be infected by siblings shedding eggs into the environment as easily as from animals. And, as you clearly stated in the introduction, none of this matters without the intermediate snail host. The model is poorly examining the obligate components of the transmission cycle (Figure 1).
  - Authors: The model was not adjusted for multiple parasitic infections among the household members as other parasitic infections in the area have different life cycles. In addition, the prevalence and intensity of infection of other helminths in the area were low.

The analysis has now been adjusted for multiple *Fasciola* infections in the household.

Available GPS data from water bodies in the properties was not reliable and for that reason not included in the analysis. No data on snails was collected for this study.

The analysis has been adjusted for reported unsafe defecation practices and water source. While the authors agree with the reviewer on the role of human in Fasciola’s lifecycle, our opinion is that in this region animals were probably more important. The authors also agree in that our model is not complete and further studies must be pursued to include all potential factors.

- Lines 128-129. The count variable is not reported anywhere in the results. If feces counts for each species are used to code species infection status variables also included in the model, these needs to be described.
  - Authors: In the multivariate model we included the overall count of animal feces inside each radius and the individual species sample infection status (cattle positive stools, cattle negative stools, sheep positive stools, sheep negative stools, pig positive stools, pig negative stools) using the household infection status as the dependent variable. Variables that were discarded from the model are now reported under the supplemental material. We used a p value < 0.2 for variable retention in the regression model.

All models are now adjusted by safe water practices, defecation practices, altitude of the household, and multiple infections within a household.

We have included under the different radius areas the number of stool samples, to estimate effect size. Please see the supplementary material now included with the manuscript.

- Lines 131-136. This is not an adequate description of the cluster analysis intent and method. What specific relationships are you probing to define as hot zone or cold zone? Jumping ahead to Figure 2, it appears your research questions include animal to animal geospatial relationships and animal to household. This all needs to be described.
  - Authors: We have expanded our spatial analysis description and clarified the methods.

**REVIEWER 2**

- Needs a little more on snail habitats
  - Authors: We did not collect data regarding snail habitats in this study.

**RESULTS:**

**REVIEWER 1**

- Line 146-147. Please also report how this sample size was achieved in terms of number of children per year, whether there were year-to-year differences in prevalence, and how often you came across multiple children testing positive in the same household. Also, “households” should be singular.
  Table 1. Age is reported as mean (SD) while other quantitative variables with a potentially normally distributed distribution like altitude and poverty score are in median (IQR). Did these two variables in particular not follow a Gaussian distribution?
  - Authors: This is cross sectional study within a larger cross-sectional study that took place between August of 2013 and August of 2018 in three different districts of Peru. Each community was tested once during the study and the authors do not have a way to tell if there were variations in prevalence/incidence of Fasciola in this communities.

The variables reported as median with IQR did not have a normal distribution by Shapiro-Wilk test.

- Lines 161-165. Were there any differences in types of animals by geographic district that might occur for example by clusters of livestock production sectors?
  - Authors: Animal distribution by location was added to the results.

- Line 165-166. The statement “Most feces samples were collected in the Ancahuasi district and more samples were positive in this district” does not accurately reflect what is shown in Table 2. There are extremely minimal differences in number of samples collected and positivity rate between Ancahuasi and Anta.
  - Authors: The authors agree with the reviewer. The sentence: “Most feces samples were collected in the Ancahuasi district, and more samples were positive in this district” was changed to: “Similar number of feces samples were collected from Ancahuasi and Anta, with minimal differences in positive rate”.
- Table 2. There is an “n” missing in “Total (N = 2420)(%)”. The Table header could be made clearer by putting either the (total N) or the n (%) on its own row.
  - Authors: The table has been revised.
- Lines 170-182 and Table 3. I believe this analysis is missing a final step for interpretability. Individual tests for differences in distance by positive or negative animal status and household outcome is confusing in presentation and resulting in non-interpretable significant associations. Closer scrutiny of the distances shows that households with a fasciola positive child are more likely to be close to cattle and swine feces, regardless of whether the animal feces itself was positive for the parasite, with sheep going in the opposite direction. The lack of statistical association in the animal-negative comparisons is mostly likely limited power to detect differences due to small sample sizes in the household-positive group, although the lack of reporting on group size makes this difficult to tell. A better way to do this would be to estimate the effect of distance to the animal feces sample on household positivity status, then test for effect modification by animal stool sample testing status. If the relationship between distance and outcome is modified by animal stool testing status, then report sub-group effects. Or, calculate the sub-group conditional probabilities for increases or decreases in distance on household status and the risk ratio for the probability of positive household status where the animal stool contains parasites versus where it does not. For cattle and swine, you will likely see no impact of animal feces positivity on the distance to outcome relationship, although sheep might be interesting. Be sure to adjust these models for altitude and (see above) presence of other positive children in household and water bodies.
  - Authors: Added to limitations: lack of information on the water bodies distance

The logistic regression analysis of the effect of the distance to the closest animal feces on the household infection status (adjusted for multiple infections in the household and altitude) showed a significant association (OR 0.997 95% CI 0.995-1.000, p= 0.0174). The effect modification on the model by the distance to the closest feces by species of livestock demonstrated no significant relationship to the household infection status (p=0.7053). Similarly, the effect of distance to closest feces and animal status on the distance to the closest stool was not statistically significant (p=0.9384).

- Table 3. Report your sub-group sizes and be consistent in presenting data. Either always do negative then positive feces or vice a versa. Similarly, your species-specific positives and negatives are not organized in any specific way, much less aligned in flow with the way material is presented in the results text.
  - Authors: Table 3 has been modified, negative then positives, afterwards cattle/swine/sheep as the text.

Unable to specify the subgroup sizes, as the software allows for multiple interactions with the stool as distance is being measured. This is based on the 2070 households being analyzed and the 2420 livestock feces obtained. For example, the same sheep stool might be the closest stool to two different households.

- Line 195. Should be “Table 4”. Line 189-204 and Table 4. I like the idea of this spatially comprehensive analysis approach more than the oversimplified analysis of median distance with one animal feces sample, as commented on above. However, you have not yet demonstrated the value of this multi-animal stool assessment approach by describing overall count or proportion of positive animal stools out of total counted within 50, 100, or 200 for positive and negative households. Another approach if your total counts are low is to use ratio of positive to negative at <50 meters, and <100 meters, and <200 meters. This could be put in a table in supplemental materials, but is necessary for defending exposure variable validity and for explaining why you simplified what you said you modeled (“overall count of animal feces” and “the individual species sample infection status”) vs what you report (sheep positive, cattle positive, swine positive). For that matter, I cannot figure out how the effects for the variables
  reported relate to the variables included in the model. A second point on the analysis method is that on lines 124-126 you stated you were using a backward selection modeling process. Backwards selection suggests you were seeking model parsimony, rather than fully adjusted effects. If that is the case, you must describe whether variables were removed, in what order, and how much that improved model fit. Considering you were only starting with the 2 variables above and altitude, I am not clear what the purpose for backwards selection was.
  - Authors: The effect sizes were included under supplemental material

We have re-done the analysis on the logistic regressions based on the buffer size, now all data shown is adjusted by sanitation, safe water availability, multiple household members infected with fascioliasis, altitude of the residence. We have included under the supplemental material the purpose of backward selection, the order in which the variables were removed and how that improved the model fit.

We have also determined the effect of altitude on our analysis, by re-doing logistic regression models based on different altitude strata (higher or lower than the mean). The manuscript results have been updated with this new analysis.

- Table 4. (1) Please reorder the types of animals so that they are consistent across each distance unit, either Swine, Sheep, then Cattle, or Sheep, Swine, then Cattle. The 200 meter comparison lacks Sheep positivity altogether with no explanation. (2) The title is not descriptive of the analysis, which assessed the likelihood or odds of household positivity status by proximity to different types of domesticated animal vectors. (3) The table must be free standing, meaning when you say “multivariable”, you need to clarify in the footnote what variables were included in the model, either as exposure variables or interest or confounders.
  - Authors: Table has been revised as suggested.

- Table 4. Please clarify what you mean by using household GPS where animal stool gps data was missing and how this relates to the (more statistically appropriate) statement on line 122 that “Livestock feces without GPS data were excluded from the buffer/radius analysis”.
  - Authors: In order to increase our power, when doing radius analysis, those feces from livestock that were picked up around the house with missing any GPS data (as they were not going to be located too far from the house, given our logistics) we have decided to include in the analysis by using the GPS of the house as a proxy. On the contrary, when near distance analysis was done, as location of the livestock feces is important, on the analysis we have decided to exclude the feces that did not have any GPS data of where they were located.
- Lines 208-214 and Figure 2. Define high-high, low-low, etc. in methods and Figure footnotes, and be sure to provide some context to help readers interpret what clusters of these mean in the figures. Also, you cannot cite Figure 2B before 2A; either flip your pictures or your text so 2a comes first.
  - Authors: High-high and low-low analysis have been described more extensively in the methods section.

**CONCLUSIONS:**

**REVIEWER1**

- Lines 240-243. Per my comments above about weaknesses in the analysis approach, this conclusion is not yet supported by the evidence.
  - Authors: Conclusions have been revised based on the new analysis.

- Lines 279-281. Where is this information? Any sort of assessment about location of water bodies that could harbor the snail vectors would address my comment about this being lacking as an adjustment factor in the analysis.
  - Authors: This comment has been explained before. There is a lack of waterbodies information given the nature of the study, but we have attempted to adjust for safe water and sanitation practices.
- Lines 281-284. Please explain contextual situation in Cusco further to explain how poverty in safe drinking water and sanitation would facilitate completion of the Fasciola life cycle.
  - Authors: The cycle could be further explained by the lack of sanitation, which could further contaminate the environment, as well as the lack of safe water access to drink or to clean cooking and eating supplies. In Cusco, residents are taught to boil the water or use chlorine and other water treatment techniques. However, our experience in the field has taught us that these practices are rarely followed, despite surveys showing good adherence to water boiling to prevent infections.
- Lines 288-295. The limitation should also acknowledge that analysis did not account for the role of other household members as influences on the index child’s infection status, nor the dependency of any human to human or animal to human transmission on availability of intermediate snail vectors. Directionality cannot be inferred from this cross sectional study design, and it is also possible that children could be sources of infections in the animals in their community. Adjusting for household conditions like use of latrines/toilets would have improved such inference.
  - Authors: We have adjusted by latrine use type in our final model, as well as multiple household infected and use of safe water (municipal pipes), as per request of the reviewer. Our study is nor powered nor designed to establish directionality. However, given the difference between infection rates between livestock and humans (31% vs 6% approximately in livestock vs humans respectively) it is more likely that the environmental contamination from feces of livestock have an impact on the human infection and not vice versa.

**REVIWER 3:**

- Conclusions pose doubts because of methodology and overlook of factors.
  Limitations section to be widely extended.
  Authors discussion needs improvements.
  Public health relevance is addressed.
- Authors: See answers to other reviewers

**OVERALL COMMENTS:**

**REVIEWER 1**

- A geospatial analysis of proximity to animal feces with parasites on human infection is valuable. The biggest issue is that while authors used rigor in confirming Fasciola infection in a large subset of children and local animals, they did not apply such rigor to measuring and analyzing all required conditions for Fasciola transmission. This lack in comprehensive analysis was surprising given the acknowledgement of the complex life cycle in the introduction and Figure 1. The dependency of Fasciola on a intermediate host for maturation and infectivity means that all relationships between infected and susceptible hosts is by default conditional on the host presence, and therefore the mechanism underlying and validating any relationships between animal feces and households with infections is unclear. A vague reference in discussion to location of water bodies suggests this ecological data might be available to authors but the manuscript as it stands does not suggest proof of mechanistic relationships can be resolved. Another major issue is that while multiple within-household infections was considered at some point in study design, it was not considered important as a confounder in the detection of Fasciola among siblings and nearby domestic animals and when and why multiple children in the household were tested is unclear. This may be a point that authors can easily address through adjustment of the analysis approach and would improve the interpretability of the results. Last, the methodological section suggests authors could greatly improve analysis by seeking council from a biostatistician or epidemiologist.
- Authors: Our intent was directed to show the importance of environmental contamination with livestock feces containing Fasciola eggs and how it relates (not causes) human infection. We agree that the lack of snail information, as well as, environmental and KAP information is a limitation of this study. We have expended the limitations section. We have adjusted our study by multiple infections in a household.

This is an exploratory study on the importance of environmental contamination from livestock’s feces in human Fasciola transmission.

REVIEWER 3

- This manuscript has the purpose of evaluating the spatial relationships between fascioliasis infection in children and the environmental burden of Fasciola eggs passed by different livestock in the Cusco region of Peru, that is the presence of liver fluke eggs on fecal samples collected on the soil.
  The manuscript includes many methodological problems, the baseline knowledge on the scenario is clearly insufficient regarding key factors, and there are several misinterpretations of basic concepts of the disease. Nevertheless, the originality of the approach merits an effort in the way for the needed improvement.
  1.- The first general consideration refers to the literature used. One may conclude that authors consider that human fascioliasis follows similar transmission and epidemiological characteristics everywhere. This disease is markedly heterogeneous in epidemiological facies. So, using examples from Nepal, Pakistan, Egypt, Iran, Ghana, Zambia, Indonesia, Denmark, Switzerland, or Malaysia  has no sense here. Fascioliasis in Andean areas differs very pronouncedly from fascioliasis in the afore mentioned countries. So, the literature should be reviewed and articles referring to fascioliasis in maximum South America used, above all concerning altitude areas in Andean zones.
- Authors: We have modified the references, but kept a few European studies to illustrate the differences in epidemiological facies mentioned by the reviewer.
- 2.- The second problem concerns the lack of knowledge, or at least the overlooking of key epidemiological aspects, regarding the study area:
  A) The origin of human infection is mainly through contaminated vegetables, predominantly freshwater plants, but also drinking of natural water or beverages made with sylvatic plants and natural water. In all the human endemic areas in the Andes, the infection of children occurs mainly (i) along the way to and from the school, (ii) when accompanying livestock for grazing and drinking in freshwater collections, and (iii) at home when eating contaminated plants and drinking natural water collected outside. This means that taking the household as the reference for such an analysis is not appropriate, i.e. for instance the distance from the school may be more important. B) The dependence of the rural inhabitants from natural water sources lead them to choose places for their dwelling located close to freshwater collections. These freshwater collections used to be inhabited by lymnaeid vectors and to be visited by livestock for drinking. So, these foci act for the infection of both humans and animals. C) In these rural areas, livestock moves freely around. Fences are not used, so that cattle, sheep and pigs use to appear mixed in the same grazing area. And the image one gets from a given place may differ one day from another, and one season from another Thus, no sense to compare with countries where animals are strictly controlled in farms, livestock maintained within fences, and separated according to species. D) Rural inhabitants give, however, more importance to cattle because of milk, meat and even family prestige inside the community. This means that the type of ownership by each family is a crucial factor to be considered. The infection risk in a household of a big owner may be different from that of a small owner. The following two papers may help in showing the aforementioned aspects of disease heterogeneity in humans worldwide and its complexity in the neighboring Bolivia:
  Angles R. et al., 2022. One Health action against human fascioliasis in the Bolivian Altiplano: Food, water, housing, behavioural traditions, social aspects, and livestock management linked to disease transmission and infection sources. Int. J. Environ. Res. Public Health 19, 1120, 44 pp.
  Mas-Coma S. et al., 2018. Human fascioliasis infection sources, their diversity, incidence factors, analytical methods and prevention measures. Parasitology 145 (13, Special Issue): 1665-1699.
- Authors: references were reviewed and added as suggested by the reviewer.
- 3.- Lines 47-48: "The factors associated with transmission of the disease among humans have not been well described." This is no true. Add "... in the area studied." or similar.
- Authors: The text was added as suggested.

- 4.- Lines 74-77: "However, studies in areas of the world with lower prevalence of infection have reached different conclusions.[11] A study in Qena, Egypt showed an animal prevalence ranging from 17.2 to 33.7%, but no evidence of human fascioliasis in the same region.[12]." This has no sense here. Just for information: human fascioliasis concentrates in Lower Egypt, that is, in the widely irrigated Nile Delta, where G. truncatula with other lymnaeid vectors are present, whereas Qena is in Upper Egypt, surrounded by desert and with only R. natalensis present. In the Nile Delta, human infection intensities similar to those in the Andes have recently been found. See the following: Periago M.V. et al., 2021. Very high fascioliasis intensities in schoolchildren of Nile Delta governorates: The Old World highest burdens found in lowlands. Pathogens, 10: 1210, 20 pp.
- Authors: Thank you for the comment. The manuscript has been reviewed.
- 5.- Line 78: Differences of results between studies are not inconsistencies, but the reflection of different transmission patterns and epidemiological situations. There are many articles on that.

Authors: We agree with the reviewer about different patterns of transmission. However, in the authors opinion there are not infinite scenarios, and a common denominator should be sought.

- 6.- Figure 1: This drawing cannot be accepted and reflects a lack of expertise on fascioliasis. This should be corrected, if not there are afterwards students reproducing it in their master's theses, etc. Please correct the following: (i) eggs do not seek an intermediate host, this is for the hatched miracidium to do; (ii) the drawing of the snail represents a terrestrial snail, with eyes on the tips of  the tentacles and the shell of an helicid or planorbid; when one sees a lymnaeid, one never forgets it; (iii) illustrating a cercaria by means of a drop does not appear to be logical; please substitute the drops by simplified cercariae with their body and long tail; (iv) cercariae do not infest aquatic plants, it is up to the encysted metacercariae to attach to the vegetables.
- Authors: The lifecycle schematic from CDC has now been included in the manuscript.
- 7.- Line 114: How and where the livestock feces were collected should be specified in detail. Simply collecting them from the soil? Cattle feces are well visible, but feces from sheep and pigs may be easily overlooked. This may underlie an important bias in the results, because of inadvertently giving more weight to cattle. How was this solved?
- Authors: Feces were collected around the household where little grass or other vegetation existed. Identifying sheep and pig feces in this case was not difficult.
- 8.- Line 115: Which quality control procedure was used?
- Authors: Mention on the quality controls used in the laboratory routine has been included in the methods.
- 9.- Line 127: How were areas around each household at 50, 100, and 200-meter radius delineated. In the map with Google Earth? If in the field, how were the 200 m measured?
  - Authors: Areas are measured by a geospatial software named ArcGIS, which uses GIS and mapping as a reference. There were not measurements on the field. The distances were calculated from data obtain by GPS coordinates.

- 10.- Line 137-138: The numbers and dates of the official approval documents should be added.
  - Authors: Not deemed necessary as they were specified in prior manuscripts, and this is a subanalysis of the original study.
- 11.- Table 1: It needs clarifications to be added to the legend. For instance, parentheses sometimes seem to include means, but in other cases they refer to ranges.
  - Authors: Clarification was added under the subheadings.
- 12.- Table 3: Something does not fit well here. Positive feces in sheep and pig are clearly higher than positive feces in cattle. This contradicts the conclusion of the manuscript.
  - Table 3 looks into the median distances not feces sample counts.
- 13.- Figure 2: There is no way to know which color of the circles concern cattle, sheep and pigs. Subfigure C has no sufficient resolution.
  - Authors: The resolution of the figures have been improved. The outlier analysis is based on all the animal stools and all human/animal stool collected. The large sample size allows for more accurate results, hence the reason why distribution accounting for each stool was not represented.

Subfigure class C is only meant to provide the reader a location reference, we have made the image slightly larger.

- 14.- Figure 3: Similar interpretation problem as in Figure 2.
  - Authors: Modifications were made as above.

- 15.- Lines 243-245: More manure per cow does not mean a higher number of liver fluke eggs, but eggs more diluted.
  - Authors: The authors disagree with the reviewer. The number of fluke in a cow liver is significantly larger that the number of flukes in sheep liver. Therefore, cattle are capable of passing more eggs overall than sheep.

- 16.- Line 251: Add "in the Cuzco area" or similar. In the Bolivian Altiplano, all these aspects are already well elucidated.
  - Authors: The change was made as suggested.
- 17.- Lines 264-265: "Infected animal feces were significantly closer to infected households as compared to non infected stool, especially cattle and swine feces." This means that dwellings are located close to freshwater collections, as in the Altiplano.
  - Authors: This analysis has been changed following suggestions by other reviewers.
- 18.- Lines 276-279: Delete these examples about Denmark and Switzerland. Situations are not comparable.
  - Authors: As requested, a comment was added to the manuscript. “There is a lack of fascioliasis spatial analysis data in South America, extrapolation of data from other European locations might not be appropriate given differences in epidemiological factors. For example, spatial analysis done on Danish cattle herds showed that the presence of streams, wetlands and pastures had a significant association with the presence of livestock infection.[28] While a study based in Switzerland, demonstrated that cattle were most likely infected in streams as they correspond to areas where the snail prevalence is higher.[29]”
- 19.- Lines 282-284: Again, for the same reason, delete the example of Malaysia, which moreover deals on intestinal monoxenous helminths. Same with the example of Iran. Use the aforementioned reference of Angles et al. on the Altiplano to refer to the link with the socioeconomic status. There is also an article on that aspect in Cajamarca - see in references of Angles et al.
  - Authors: The reference were added as suggested.
- 20.- Lines 288-295: This section is a good place to again refer to what has been noted above in points 2 A-D, although these crucial aspects should be referred to at least in the Discussion.
  - Authors: The references were added as suggested.
- 21.- Lines 297-298: Add "... eggs shed by cattle, sheep and goats" to avoid confusion with human stools. Outdoor defecation by rural inhabitants is commonly practiced in the Andean altitude areas.
  - Authors: No human stool was collected from the field around the households. We have added a better description of the sample collection methods for human.
- 22.- Line 301: Change "need" to "needed".
  - Authors: The change was made as suggested.
- 23.- Line 302: Change "surveillance" to "control measures".
  - Authors: The change was made as suggested.
